# Integrated multi-omics reveals distinct non-volatile and aroma signatures in albino, yellow, and purple tea varieties

**DOI:** 10.1016/j.fochx.2025.102830

**Published:** 2025-07-26

**Authors:** Yanlin An, Lihong Zhang, Xueqi Li, Xiaozeng Mi, Dahe Qiao, Tingting Jing

**Affiliations:** a School of Food Engineering, Moutai Institute, Luban Street, Renhuai 564502, Guizhou, China; b State Key Laboratory of Tea Plant Germplasm Innovation and Resource Utilization, Anhui Agricultural University, 130 Changjiang West Road, Hefei, China; c Guizhou Tea Research Institute, Guizhou Academy of Agricultural Sciences, Guiyang, Guizhou 550006, China

**Keywords:** Tea of different colors, Non-volatiles, Anthocyanin, Amino acid, Volatiles, Aroma

## Abstract

Non-volatile metabolites and aroma substances are important quality components of tea plants, yet the quality characteristics of tea plants with special leaf colors still await further revelation. In this study, by integrating five analytical methods including widely targeted metabolomics, HS-SPME-GC–MS, and targeted metabolomics, a total of 1770 non-volatile metabolites were detected in ZK, NB, and ZH3, among which flavonoids, amino acids, and polyphenols were the main differential metabolites. The contents of total catechins, anthocyanins and amino acids in ZK, NB and ZH3 are 159.96, 122.03, 121.86 mg/g, 4.22, 4.77, 4.70 mg/g and 22.27, 60.01 and 61.83 mg/g, respectively. A total of 122 volatile compounds were identified, mainly including 36 alcohols, 20 aldehydes, 16 esters and 16 ketones. And 41, 36 and 37 key aroma compounds were identified in NB, ZH3 and ZK, respectively. These results provide valuable insights into understanding the quality basis of tea plants with different leaf colors.

## Highlights

• 1770 non-volatile metabolites were identified in three tea trees through the widely targeted metabolome.
• The contents of total catechins, caffeine and theobromine in ZK are significantly higher than those in ZH3 and NB.
• The total amino acid content in ZH3 and NB is more than twice that in ZK.
• The contents of most anthocyanin components in ZK are significantly higher than those in ZH3 and NB. 5. 122 volatile aroma compounds were identified, of which 41, 36 and 37 key aroma compounds were found in NB, ZH3 and ZK.

## 1 Introduction

Due to its rich content of elements beneficial to human health, such as amino acids and flavonoids, tea (*Camellia sinensis*) has become the most popular green beverage and is widely consumed worldwide (Jin et al., 2023). China, as the country that was the first to cultivate and utilize tea plants, harbors the most abundant tea plant resources. There are more than 600 cultivated tea plant varieties alone. These rich genetic resources provide immense potential for tea genetic breeding and the development of tea beverages (Chen et al., 2024; Kong et al., 2025; J. Li et al., 2024). In many previous reports, extensive research has been conducted on some special tea varieties with characteristics such as low caffeine content, high amino acid content, and high anthocyanin content. Due to their low chlorophyll content or rich carotenoid and anthocyanin content, these tea plants tend to exhibit albinism, yellowing, and purpling traits (Xie et al., 2025). Teas crafted from the tender leaves of these tea plants often feature distinctive flavor profiles and unique health promoting properties (L. Liu et al., 2024).

Non-volatile metabolites are important components of the taste of tea soup (Y. Wang, Fang, et al., 2021). People have devoted a great deal of effort to qualitatively and quantitatively determining these substances in the hope of deeply understanding the basis of tea quality formation (J. Li et al., 2024). Usually, albino tea plants are divided into two types: temperature-sensitive varieties and light-sensitive varieties, and their underlying genetic regulatory mechanisms are different (Liu, Xu, et al., 2023; W. Zhu et al., 2024). A comparative analysis has found that the levels of amino acids and flavonoids in the leaves of albino tea plants are significantly different from those in green leaves. Moreover, catechins and carotenoids are considered to be the most important contributors to the metabolic characteristic differences between albino and green tea varieties (Chen et al., 2024). Unlike albino tea, yellow tea leaves not only contain a relatively high level of amino acids but also accumulate more carotenoids (Cheng et al., 2019). However, there is still relatively little research on yellow tea leaves at present. For purple leaf tea, its core feature is that the content of anthocyanins can reach dozens of times that of ordinary green tea, giving the tea soup a purple-red color. In addition, the contents of catechins, flavonol glycosides and proanthocyanidins were significantly higher than those of ordinary green tea, which together contributed to strong antioxidant activity (Yang et al., 2025). Due to the great health value of anthocyanins, in recent years, many studies have deeply revealed the synthesis and genetic regulatory mechanisms of anthocyanins in purple leaf tea plants from different perspectives (Xie et al., 2025).

Volatile metabolites are another important criterion for evaluating the quality of tea (J. Li et al., 2024). Currently, more than 700 types of volatile metabolites have been identified in tea, and together they endow and shape the pleasant aroma of the tea soup (Zhai et al., 2022). Among these metabolites, there are a large number of substances such as alcohols, aldehydes, ketones, and lipids (J. Li et al., 2024). For example, Kun identified a total of 81 volatile metabolites in five green teas with different aroma types. Among them, the numbers of alcohols, aldehydes, ketones and lipids were 17, 15, 10 and 6 respectively (Qin et al., 2024). While, Qin et al. identified 173 volatile components in steamed green tea, and eight substances including dimethyl sulfide, (*E*)-β-ionone, cis-jasmone, linalool, nonanal, heptanal, isovaleraldehyde and (Z)-3-hexenol were regarded as key aroma components (Qin et al., 2024). By comparing four different white tea varieties, it was found that 2-ethyl-3,5dimethylpyrazine, 2,3-diethyl-5-methylpyrazine and dimethyl sulfide contributed to the formation of ripe corn aroma and roasted aroma of tea (Y. Li et al., 2025); According to the results of extensive aroma analysis, it is found that in white-leaf tea, the contents of nerol, *Z*-isogeraniol, and E-3-hexen-1-yl acetate have increased, while the contents of β-myrcene, benzyl alcohol, and methyl salicylate decrease in purple-leaf tea (F. Wang et al., 2025). In conclusion, numerous studies have comprehensively revealed the taste components and aroma characteristics of different types of tea, and found that there are significant differences in multiple aspects among teas with different leaf colors (W. Huang et al., 2023; Ye et al., 2024). However, regarding some teas with special colored leaves, their quality characteristics have not been reported yet, and there is also a lack of unified and comprehensive comparative studies.

In this study, we selected three teas with different colors, namely NaiBaiCha (NB, a albino variety), ZhongHuang 3 (ZH3, a yellow-leaf variety), and ZiKui (ZK, a purple-leaf tea), as the research objects. Through multi-omics analyses such as widely targeted metabolomics, SPME-GC–MS, amino acid and anthocyanin quantification, we comprehensively interpreted their quality characteristics and key differential components. In conclusion, this study helps to deepen our understanding of the characteristic metabolites such as taste and aroma in tea tree varieties of different colors under a unified scale.

## 2 Materials and methods

### 2.1 Plant material

The one-bud-two-leaf samples of three tea tree cultivars, NaiBaicha (NB), ZhongHuang 3 (ZH3), and ZiKui (ZK), were collected from the Meitan Experimental Base (27.44′N, 107.27′E) of the Tea Research Institute, Guizhou Academy of Agricultural Sciences. The collected samples were spread out in a well-ventilated room at room temperature for 6 h. During the spreading process, the tea leaves were turned over 2–3 times to maintain humidity between 75–85 %. Subsequently, the samples were subjected to the fixing process at 260 °C for 3 min. After that, the fixed samples were allowed to absorb moisture at room temperature for 2 h and then rolled for 40 min. Finally, all samples were dried in an aroma-enhancing machine to ensure their moisture content was below 6 % (J. Li et al., 2024).

### 2.2 Sample preparation and detection of non-volatile metabolites

For sample preparation, 50 mg of each sample was weighed and extracted with 1000 μL of extraction solution (methanol:acetonitrile:water = 1:2:1, *v*/v/v). Non-volatile metabolites were detected using a UPLC-ESI-MS/MS system (UPLC: Waters Acquity I-Class PLUS; MS: Applied Biosystems QTRAP 6500+). UPLC Conditions: Column: Waters HSS-T3 (1.8 μm, 2.1 mm × 100 mm). Mobile phases: solvent A: pure water containing 0.1 % formic acid and 5 mM ammonium acetate, solvent B: acetonitrile containing 0.1 % formic acid. Gradient program: Initial: 98 % A, 2 % B (held for 1.5 min); 5.0 min: Linear gradient to 50 % A, 50 % B, 9.0 min: Linear gradient to 2 % A, 98 % B (held for 1 min); Post-run: Return to 98 % A, 2 % B in 1 min (held for 3 min). The operation parameters of the ESI source are as follows: the source temperature is 550 °C; the ion source gas I (GSI), gas II (GSII), and curtain gas (CUR) are set at 50, 55, and 35 psi respectively; the collision-activated dissociation (CAD) is set to medium. For the QQQ and LIT modes, 10 μmol/L and 100 μmol/L polypropylene glycol solutions are used respectively for instrument tuning and mass number calibration. The QQQ scans are obtained when the collision gas (nitrogen) is set to medium in the MRM experiment. The declustering potential (DP) and collision energy (CE) for individual MRM transitions are determined through further optimization of DP and CE. According to the metabolites eluted during each period, a specific set of MRM transitions for that period is monitored. For more detailed methods and descriptions, please refer to our previous research (L. Liu et al., 2024).

### 2.3 Quantitative detection of catechins by HPLC

To prepare the sample, first weigh 50 mg of dried tea sample and place it in a centrifuge tube, add 4 mL of 80 % methanol solution for mixing, and then shake ultrasonically for 30 min under ice bath conditions. After sonication, centrifuge at 13,000 rpm for 10 min, aspirate 1.5 mL of centrifuged supernatant and filter it using a 0.22 μm organic membrane, and finally transfer the filtered liquid into the injection bottle. Meattime, Gallocatechin (GC), epigallocatechin (EGC), catechin (C), epigallocatechin-3-gallate (EGCG), epicatechin (EC), epicatechin-3-gallate (ECG), and caffeine were used as standards for sample quantification. The peaks were identified by comparing the retention times of the samples with those of the standards. For more detailed elution conditions, please refer to Liu et al.'s previous studies (S. Liu et al., 2019).

### 2.4 Quantitative detection of amino acids by UHPLC-MRM-MS/MS

Add the weighed sample into a 2 mL centrifuge tube, then add 1 mL of a solution consisting of methanol, acetonitrile and water in a volume ratio of 2:2:1 (*V*/V/V) and vortex for 30 s. Then, homogenize and shake the mixture at 45 Hz for 4 min, and conduct ultrasonic treatment for 5 min under an ice-water bath condition. Repeat the steps of homogenization and ultrasonic treatment for 3 times. After standing at −20 °C for 1 h, centrifuge it at 12,000 r/min for 15 min under the condition of 4 °C. Finally, take 200 μL of the supernatant, filter it through a 0.22-μm filter membrane, and then inject it for analysis. The 37 kinds of amino acids in tea tree samples were detected using a Waters ACQUITY I-Class ultra-high performance liquid chromatograph. The target compounds were chromatographically separated by an ACQUITY UPLC BEH Amide (150 × 2.1 mm, 1.7 μm, Waters) liquid chromatography column. The mobile phase A of the liquid chromatography was an aqueous solution containing 0.2 % formic acid, and the mobile phase B was acetonitrile containing 0.2 % formic acid. The temperature of the column oven was set at 35 °C, the sample tray was set at 10 °C, and the injection volume was 1 μL. In this project, a SCIEX QTRAP 6500+ triple quadrupole mass spectrometer equipped with an IonDrive Turbo V ESI ion source was used, and mass spectrometry analysis was carried out in the multiple reaction monitoring (MRM) mode. The ion source parameters are as follows: Curtain Gas = 35 psi, IonSpray Voltage = +5500 V, −4500 V, Temperature = 550 °C, Ion Source Gas 1 = 50 psi, Ion Source Gas 2 = 55 psi. Before performing UHPLC-MS/MS analysis, the standard solutions of the target compounds were introduced into the mass spectrometer. For each target compound, several parent ion-daughter ion pairs (transitions) with the highest signal intensity were selected, and their MRM parameters were optimized. Among them, the ion pair with the best response was selected for quantitative analysis, and other ion pairs were used for the qualitative analysis of the target compounds. All mass spectrometry data collection and quantitative analysis of the target compounds were completed through the SCIEX Analyst Work Station Software (Version 1.7.2) and Sciex OS 2.0.1 (Šimura et al., 2018).

### 2.5 Quantitative detection of anthocyanin content in tea samples

Weigh an appropriate amount of the sample into a 2 mL centrifuge tube, add 1 mL of a methanol: water solution (7:3, *V*/V, containing 0.1 % formic acid), and vortex for 30 s. Then, homogenize the sample at 45 Hz for 15 min, and conduct ultrasonic treatment for 30 min under an ice-water bath condition. Centrifuge the sample at 12,000 r/min for 15 min at 4 °C. Take 200 μL of the supernatant, filter it through a 0.22-μm filter membrane, and then inject it for analysis. Accurately pipette the corresponding amount of the standard substance (1 mg/mL) into a 10 mL volumetric flask, and prepare a mixed standard solution with a concentration of 20 μg/mL. Dilute this standard solution successively to obtain a series of calibration solutions.

Use a Waters ACQUITY I-Class ultra-high performance liquid chromatograph to chromatographically separate the target compounds through an ACQUITY UPLC HSS T3 (100 × 2.1 mm, 1.8 μm, Waters) liquid chromatography column. The mobile phase A of the liquid chromatography is an aqueous solution containing 0.1 % formic acid, and the mobile phase B is acetonitrile containing 0.1 % formic acid. The temperature of the column oven is set at 35 °C, the sample tray is set at 10 °C, and the injection volume is 2 μL. Use a SCIEX QTRAP 6500+ triple quadrupole mass spectrometer equipped with an IonDrive Turbo V ESI ion source to conduct mass spectrometry analysis in the multiple reaction monitoring (MRM) mode. The ion source parameters are as follows: Curtain Gas = 35 psi, IonSpray Voltage = +5500 V, −4500 V, Temperature = 550 °C, Ion Source Gas 1 = 50 psi, Ion Source Gas 2 = 55 psi. Before performing UHPLC-MS/MS analysis, introduce the standard solutions of the target compounds into the mass spectrometer. For each target compound, select several parent ion-daughter ion pairs (transitions) with the highest signal intensity, optimize their MRM parameters, and select the ion pair with the best response for quantitative analysis, while other ion pairs are used for the qualitative analysis of the target compounds.

### 2.6 Detection of volatile compounds in tea samples

Take 700 mg sample into the 20 mL headspace bottle, add 10 μL of 2-Octanol as internal standard; All samples were analyzed by gas chromatograph system coupled with a spectrometer (GC–MS). In SPME cycle of PAL rail system. Incubate temperature is 60 °C; Preheat time in 15 min; Incubate time in 30 min; Desorption time is 4 min.GC–MS analysis was performed using an Agilent 7890 gas chromatograph system coupled with a 5977B mass spectrometer. The system utilized a DB-Wax. Injected in splitless mode. Helium was used as the carrier gas, the front inlet purge flow was 3 mL/min, and the gas flow rate through the column was 1 mL/min. The initial temperature was kept at 40 °C for 4 min, then raised to 245 °C at a rate of 5 °C/min, kept for 5 min. The injection, transfer line, ion source and quad temperatures were 250, 250, 230 and 150 °C, respectively. The energy was −70 eV in electron impact mode. The mass spectrometry data were acquired in scan mode with the *m*/*z* range of 20–400, solvent delay of 0 min. Chroma TOF 4.3× software of LECO Corporation and Nist database were used for raw peaks exacting, the data baselines filtering and calibration of the baseline, peak alignment, deconvolution analysis, peak identification, integration and spectrum match of the peak area.Detailed methods can refer to our previous research (L. Liu et al., 2024).

### 2.7 Statistics analysis

All samples in this study were set with three biological replicates. All quantitative data were expressed as the mean ± standard deviation (SD) and analyzed using SPSS software for analysis of variance (ANOVA). Differences between means were considered statistically significant when *P* < 0.05. The construction of PCA and PLS-DA models was performed using the OmicShare (https://www.omicshare.com/) web platform and SIMCA 14.1, respectively.

## 3 Results and discussion

### 3.1 Analysis of metabolites in different samples by widely targeted metabonomics

**Fig. 1:**
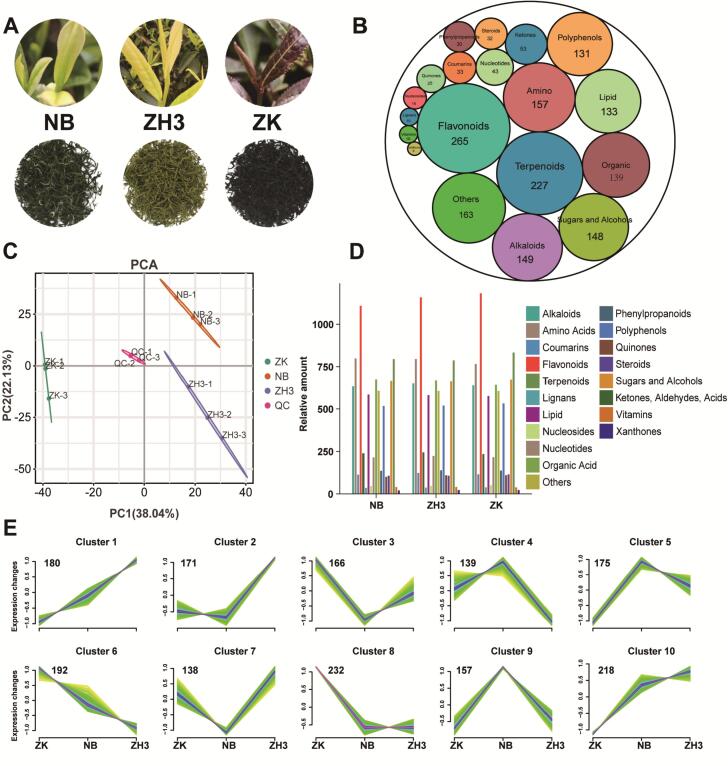
Overview of non-volatile metabolites. (A) Three samples of different colored teas; (B) Types of non-volatile metabolites; (C) PCA Analysis Based on nonvolatile metabolites; (D) Content analysis of non-volatile metabolites in different categories; (E) Content pattern cluster of non-volatile metabolites in three samples.

Non-volatile metabolites such as polyphenols and flavonoids are the main components contributing to the taste of tea soup, and their contents may vary significantly among different tea varieties (J. Wang et al., 2024). In this study, we detected a total of 1770 non-volatile metabolites from the three tea tree varieties, ZK, NB, and ZH3 (Fig. 1A). Further classification found that these metabolites were mainly polyphenols, flavonoids, terpenoids, amino acids, alkaloids, organic acids, and sugars and alcohols, and their quantities were 131, 265, 227, 157, 149, 139, and 148, respectively (Fig. 1B). PCA analysis based on these metabolites can provide a comprehensive perspective on the differences among samples. The results show that the nine tea samples are perfectly divided into three groups. Among them, ZK, NB, and ZH3 are located in three different regions on the left side, the upper right corner, and the lower right corner of the graph respectively. Meanwhile, the three QC samples are closely clustered at the center of the figure, reflecting the high reliability of our results. The interpretation rates of the first principal component and the second principal component reach 38.04 % and 22.13 % respectively (Fig. 1C). This result implies that there are significant differences in the contents of non-volatile metabolites in tea leaves of different colors. (Fang et al., 2023; T. Huang et al., 2025). The relative content of metabolites is represented by the peak area (the peak area is normalized using Log 10). As shown in Fig. 1D, flavonoids, amino acids, and terpenoids are the three types of compounds with the highest contents. Meanwhile, these metabolites can be classified into 10 characteristic clusters, and the number of metabolites contained in each cluster ranges from 139 to 232 (Fig. 1E). For example, among the 232 metabolites in Cluster 8, there are 50 flavonoid metabolites and 50 terpenoid metabolites, while there are only 12 amino acid substances. In contrast, among the 218 metabolites in Cluster 10, there are 53 amino acid metabolites. Such results reflect that metabolites of the same class may tend to have similar expression trends in different tea samples (F. Wang et al., 2025). A more detailed metabolite table can be obtained at this link: https://gitee.com/qiushui1234567/metabolomic.

### 3.2 Identification of differential non-volatile compounds

**Fig. 2:**
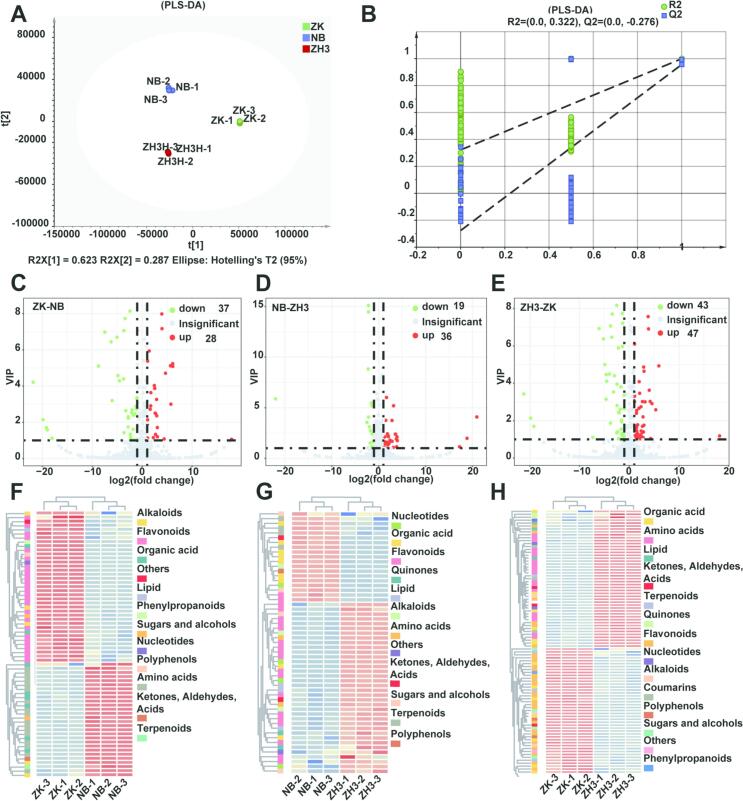
Identification of different non-volatile metabolites by PLS-DA model. (A) Scores plots of PLS-DA (R^2^X = 0.911, R^2^Y = 0.999, Q2 = 0.996); (B) cross-validation plot by 200 permutation tests (R^2^ = 0.322, Q^2^ = −0.276); (C-E) Volcano map shows the number of different metabolites of ZK vs NB, NB vs ZH3 and ZH3 vs ZK; (F-G) The differential metabolite content of ZK vs NB, NB vs ZH3 and ZH3 vs ZK was demonstrated by heat map.

In order to evaluate the differential non-volatile components in the three tea leaves of different colors, we adopted the widely-used PLS-DA model to analyze the metabolites (Shan et al., 2024; Song et al., 2025). As shown in Fig. 2A, the results of the model indicate a high level of variance (R^2^X = 0.911, R^2^Y = 0.999) and good predictive ability in cross-validation (Q^2^ = 0.996). The reliability of this model was subsequently verified through 200 permutation tests. The values of R^2^ and Q^2^ were 0.322 and − 0.276 respectively, which indicates that the model is not overfitted. Here, we identified the differential metabolites in different comparison groups by combining the VIP (Variable Importance in the Projection) values and Fold change values. In the three comparison groups of ZK vs NB, NB vs ZH3, and ZH3 vs ZK, 65 (including 28 up-regulated and 37 down-regulated), 55 (including 36 up-regulated and 19 down-regulated), and 90 (including 47 up-regulated and 43 down-regulated) differential metabolites were respectively identified (VIP > 1 and |log2(fold change)| > 1) (Fig. 2C-E) (Tan et al., 2023; Yue et al., 2023). However, the differential metabolites in the three groups are different. Previous studies have demonstrated that albino tea and yellow-leaf tea have higher amino acid contents and lower flavonoid contents (Cheng et al., 2019; W. Zhu et al., 2024). Our results also indicate that the differential metabolites in the two groups (ZK vs NB and ZH3 vs ZK) mainly include flavonoids and amino acids. However, the amino acid contents in albino tea and yellow-leaf tea are usually not significantly different (Zhu, Liu, et al., 2023). For the comparison between NB and ZH3, the differential metabolites are mainly flavonoids and organic acids (Fig. 2F-H).

### 3.3 Analysis of the contents of catechin and alkaloids in the sample

**Fig. 3:**
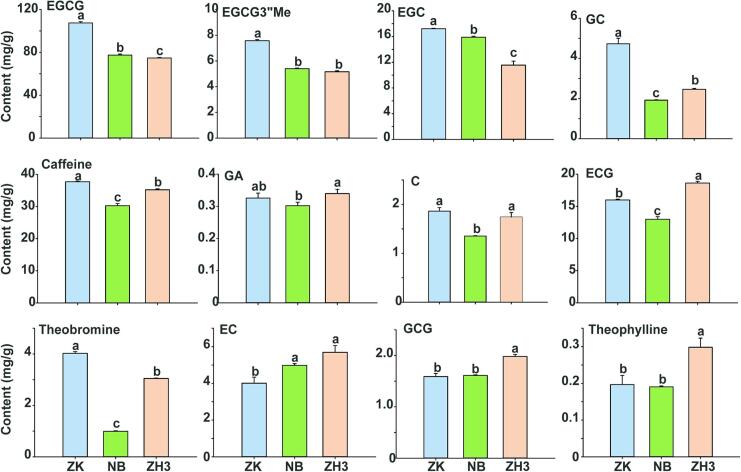
The contents of catechin, caffeine, theobromine and theophylline in three tea samples were quantitatively detected by HPLC.

Flavonoid compounds are secondary metabolites of plants, which are widely distributed in the plant kingdom and possess strong biological activities. Catechin is one of the main flavonoid compounds, and its content accounts for 12 % to 25 % of the dry weight of fresh tea leaves (Y. Wang et al., 2019). The quantitative results showed that the total catechin contents in the ZK, NB, and ZH3 samples were 159.96, 122.03, and 121.86 mg/g, respectively. The quantitative results showed that the total catechin contents in the ZK, NB, and ZH3 samples were 159.96, 122.03, and 121.86 mg/g, respectively. Among them, the contents of five kinds of catechins, namely EGCG, EGCG”Me, EGC, GC and C in ZK were all higher than those in NB and ZH3 (Fig. 3). At the same time, the contents of caffeine and theobromine were also significantly different among ZK, NB and ZH3, and their contents reached 37.64, 30.45, 35.20 and 4.03, 1.00 and 3.05 mg/g, respectively. High concentrations of catechins and alkaloids may be an important reason for the more bitter and astringent taste of the ZK tea soup (X. Huang et al., 2022). In addition, the content of GA (gallic acid) in NB tea was the lowest, while the content of theophylline in ZH3 tea was the highest.

### 3.4 Compare the differences in the contents of anthocyanidin components

**Fig. 4:**
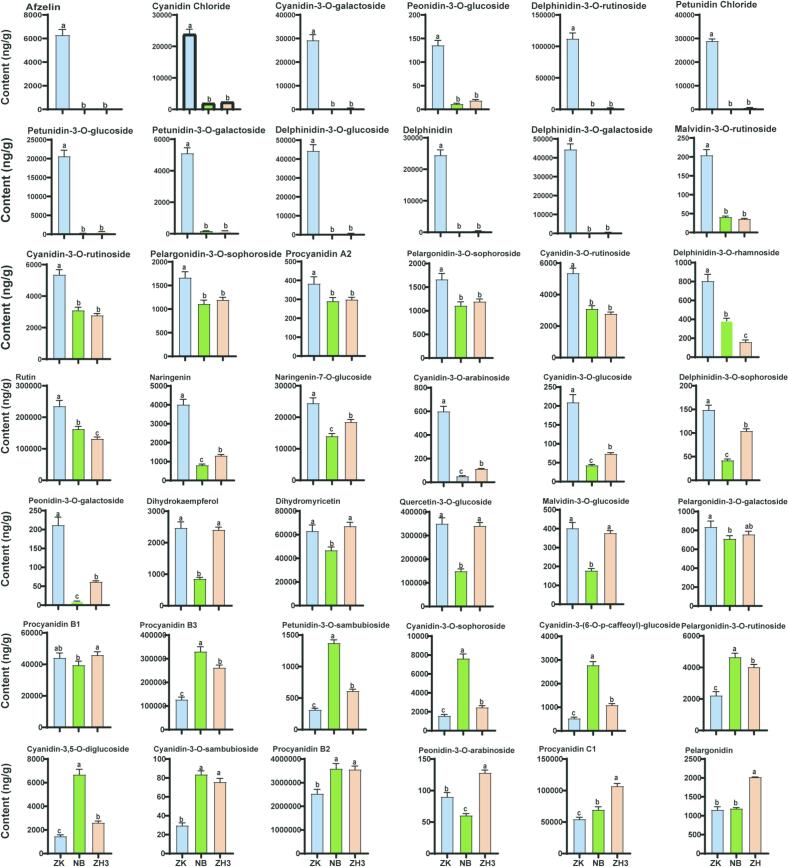
Detection of anthocyanin components in three tea samples by targeted metabolomics.

**Supplementary fig. S1:**
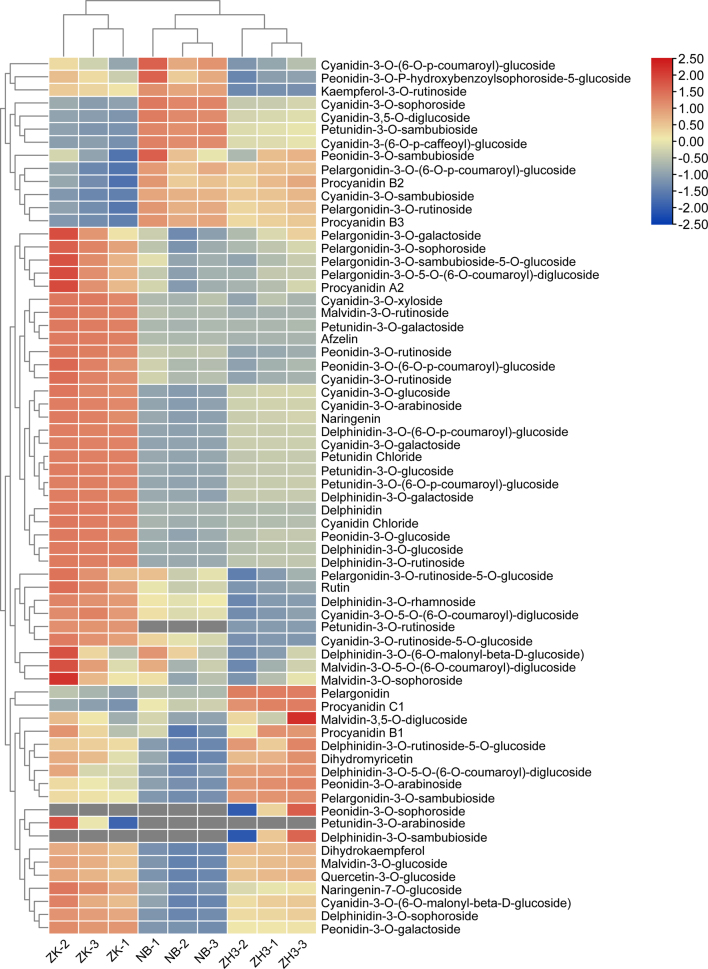
Heat map of anthocyanin content in three tea plant samples of different colors.

**Supplementary fig. S2:**
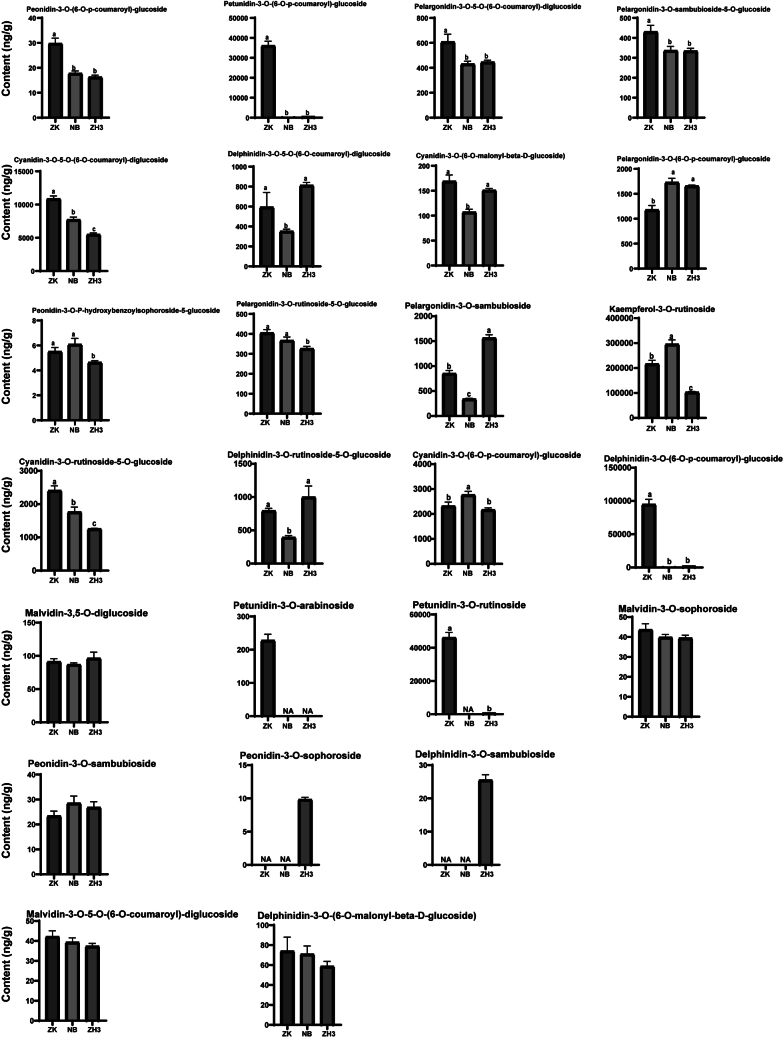
Graph of partial anthocyanin contents in three tea plant samples of different colors.

Anthocyanin components belong to another important category of water-soluble active substances with strong antioxidant properties among flavonoid substances. Anthocyanins in plants are mainly divided into six categories: cyanidin, delphinidin, peonidin, pelargonidin, malvidin and petunidin (Hsu et al., 2012). Studies have shown that there may be significant differences in the content of anthocyanins among different tea tree varieties. Moreover, a high content of anthocyanins can affect the color of the tea soup and enhance its bitterness and astringency (Xie et al., 2025). In this study, we identified a total of 67 anthocyanin components (Fig. S1). Interestingly, there was not much difference in their total contents among ZK, NB, and ZH3, which were 4.22, 4.77, and 4.70 mg/g respectively. However, as depicted in Fig. 4 and Fig. S2, apart from five substances including Malvidin-3,5-O-diglucoside, Malvidin-3-O-sophoroside, and Peonidin-3-O-sambubioside, which showed no statistical differences among the three samples at all, the contents of at least 32 anthocyanin/anthocyanin glycoside components in ZK were significantly higher than those in NB and ZH3. These substances with differences include Afzelin, Cyanidin Chloride, Cyanidin-3-O-galactoside, Petunidin Chloride, Delphinidin, Malvidin-3-O-rutinoside and so on. However, the contents of substances such as Procyanidin B3, Petunidin-3-O-sambubioside, Cyanidin-3-O-sophoroside and Cyanidin-3-(6-O-p-caffeoyl)-glucoside in NB tea are significantly higher than those in ZK and ZH3; the contents of substances such as Peonidin-3-O-arabinoside, Procyanidin C1, Pelargonidin-3-O-sambubioside in ZH3 are significantly higher than those in ZK and NB. Heat map analysis shows that the high contents of substances such as Procyanidin B2, Quercetin-3-O-glucoside, Procyanidin B3 and Procyanidin C1 in either ZH3 or NB greatly compensate for the differences compared with ZK (Fig. S2).

### 3.5 Reveal the differences in the amino acid contents among different samples

**Fig. 5:**
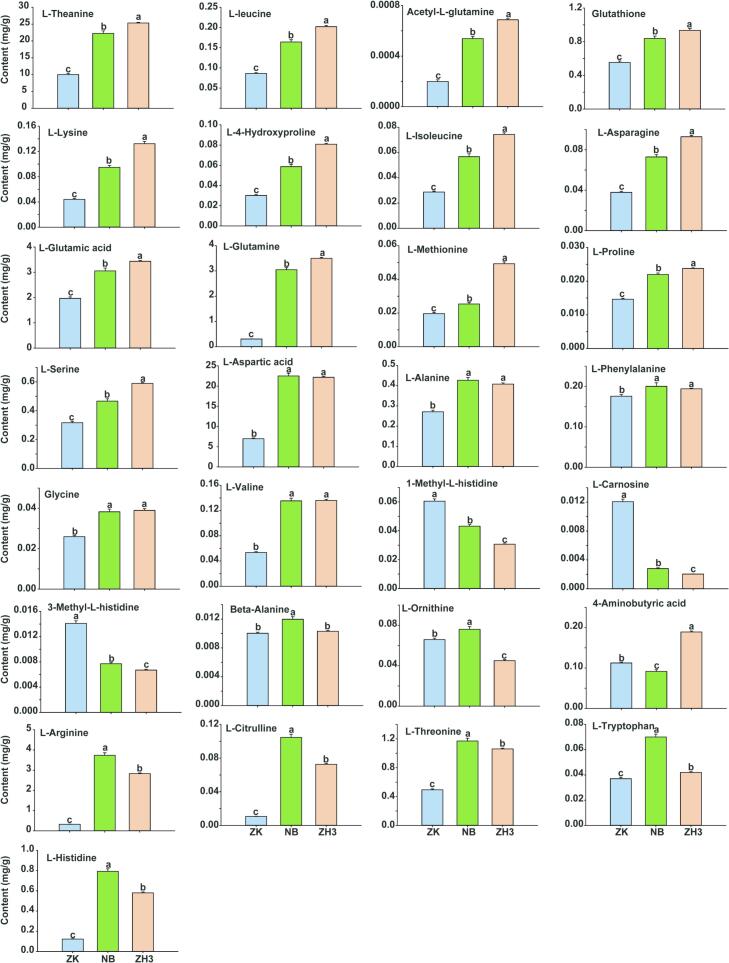
Detection of amino acids in three tea samples by targeted metabolomics.

The abundant free amino acids in tea leaves can enhance the umami taste of the tea broth and counteract its astringency and bitterness. However, the synthesis of amino acids in tea leaves is jointly regulated by multiple factors. Generally, the amino acid content in albino and yellow-leaved tea is higher than that in normal tea leaves (Cheng et al., 2019). In this study, a total of 29 free amino acids, including theanine, were detected in the tea samples. The results showed that there were significant differences in the total amino acid contents among ZK, NB and ZH3, with the values being 22.27, 60.01 and 61.83 mg/g respectively (Fig. 5). Among these amino acids, the three amino acids with the highest contents are L-Theanine, L-Aspartic acid and L-Glutamic acid respectively. As the most representative and highest-content amino acid in tea leaves, the contents of theanine in ZK, NB and ZH3 are 10.7, 22.38 and 25.11 mg/kg respectively, accounting for 48 %, 37 % and 40 % of their respective total amino acid contents. Higher catechin and alkaloid content and lower amino acid content may be the main reasons for the bitterness of ZK tea soup (Tan et al., 2023). Due to the competitive relationship between the synthesis of amino acids and flavonoids, and the abnormal development of chloroplasts in albino and yellow-leaf tea plants, light stress is reduced, which leads to the weakened synthesis of flavonoids and the retention of more amino acids. This results in a significantly higher content of flavonoids in ZK tea than in NB and ZH3, while the amino acid content is significantly lower than in the latter two (Y. Wang, Cheng, et al., 2021; Zhu, Qiao, et al., 2023).

### 3.6 Insight into the total volatile compounds in the samples

**Fig. 6:**
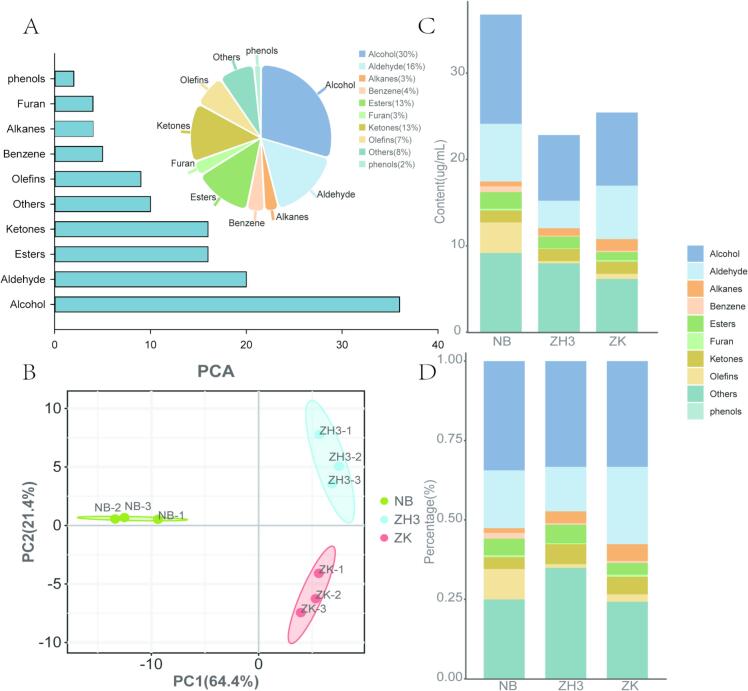
Overview of volatile compounds. (A) Classification and proportion of volatile compounds; (B) PCA analysis based on volatile compounds; (C) The stacked histogram shows the total volatile content of the three tea plant samples; (D) The percentage histogram shows the proportion of different types of volatile compounds in each tea sample.

In this study, a total of 122 volatile compounds were identified by using headspace-SPME-gas chromatography–mass spectrometry (HS-SPME-GC–MS) technique. Among them, there are 36 alcohols, 20 aldehydes, 16 esters, 16 ketones, 10 olefins, 5 benzenes, 4 alkanes, 4 furans, 2 phenols and 10 compounds classified as others (Fig. 6A). More detailed volatile components can be obtained through the following links: https://gitee.com/qiushui1234567/metabolomic. The results of the PCA model analysis based on these components showed that all the samples were well classified into three groups. The interpretation rates of the first principal component and the second principal component reached 64.4 % and 21.4 % respectively (Fig. 6B). Among the three samples, the total volatile components in NB tea were significantly higher than those in ZH3 and ZK, with the values being 36.79, 22.83 and 25.44 μg/mL respectively (Fig. 6 C). Alcohols, aldehydes, ketones and esters are the most representative aroma components, and the proportion of their contents varies among different tea tree varieties (F. Wang et al., 2025; Zhang, 2024). For example, the proportions of alcohols in NB, ZH3 and ZK are extremely similar, accounting for 34.4 %, 33.3 % and 33.3 % respectively, while the proportions of aldehydes show obvious differences, being 18.2 %, 13.97 % and 33.29 % respectively. Such differences may potentially lead to the distinct aroma characteristics of these three samples (Fig. 6 D) (Song et al., 2025; Ye et al., 2024).

### 3.7 Identification of differential volatile compounds

**Fig. 7:**
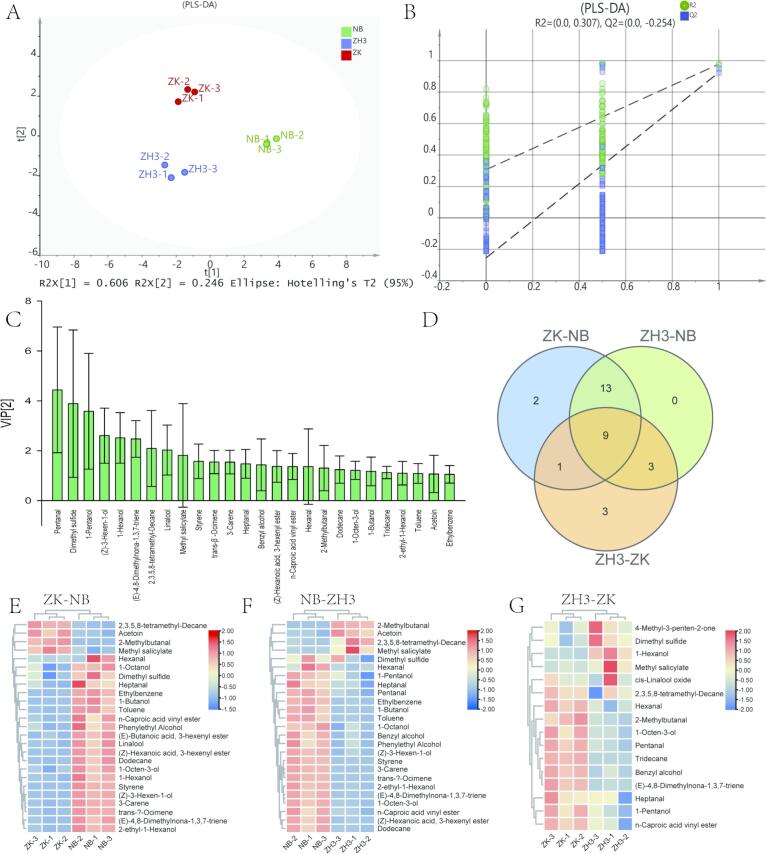
Identification of different volatile compounds by PLS-DA model. (A) Scores plots of PLS-DA (R2X = 0.852, R2Y = 0.978, Q2 = 0.955); (B) cross-validation plot by 200 permutation tests (R2 = 0.307, Q2 = −0.254); (C) Differential volatile compounds in three tea plants; (D) Venn plots show the number of differential volatile compounds in different combinations；(E) Heat maps show the amount of differential volatile compounds in different combinations.

In order to identify the differential volatile metabolites, a PLS-DA model was executed based on the contents of 122 volatile aroma compounds. The model results showed that all samples were well classified according to their different colors. For the model, R^2^X was 0.852, R^2^Y was 0.978, and Q^2^ was 0.955 (Fig. 7A). Besides, the 200-time permutation test confirmed the feasibility and reliability of the established PLS-DA model (R^2^ = 0.307, Q^2^ = −0.254) (Fig. 7B). Finally, 26 differential volatile components (VIP > 1) were identified in the samples of this study, including pentanal (almond), dimethyl sulfide (green, fresh), 1-pentanol (fruity, fusel-like), (Z)-3-hexen-1-ol (green, lettucelike), 1-hexanol (green, grassy), linalool (fruity, floral), heptanal (green, grassy), hexanal (green, grassy), 1-butanol, 2-methylbutanal (caramel, nutty) and 1-Octen-3-ol (sweet), etc. (Fig. 7C). These components have been identified as differential compounds in many studies, and they mainly contribute to the characteristics such as the fresh fragrance and the floral and fruity aroma of the tea (Hao et al., 2023; Liu, Shen, et al., 2023; Zhang et al., 2023). In fact, 25 differential metabolites were identified between the two groups of ZK vs NB and ZH3 vs NB, while only 16 differential compounds were identified between the two samples of ZH3 vs ZK (Fig. 7D). Fig. 7E-F illustrates the expression characteristics of the differential compounds in different comparison groups, in which ZK vs NB contains two unique differential compounds of (*E*)-butanoic acid, 3-hexenyl ester and 1-hexanol, while ZH3 vs ZK contains three unique differential compounds of cis-linalool oxide, tridecane and 4-methyl-3-penten-2-one.

### 3.8 Key aroma-active volatiles in three different color teas

The unique aroma of tea is influenced not only by the content of volatile compounds but also by their odor thresholds (T. Huang et al., 2025; Ma et al., 2024). The odor activity value (OAV) is an indicator used to quantify and evaluate the contribution of various volatile compounds to the aroma of tea. Typically, volatile compounds with OAV ≥ 1 are considered key aroma components of tea samples, and a higher OAV value indicates a greater influence on the aroma (Y.-L. Zhu et al., 2025). According to such criteria, we identified 41, 36 and 37 key aroma-active volatile compounds in the tea plants of NB, ZH3 and ZK respectively (Table 1). Compared with ZH3 and ZK, six volatile substances such as phenylethyl alcohol (floral), 1-hepten-3-one (roasted), 2,6,6-trimethyl-1,3-cyclohexadiene-1-carboxaldehyde (woody), 2,6,6-trimethyl-1-cyclohexene-1-carboxaldehyde (fruity), trans-β-ocimene (floral) and styrene (balsamic) can be regarded as the key aroma components unique to NB, which enhance its floral and fruity aroma as well as the roasted aroma. While 2-methylbutanal, nonanal, and (E)-2-exenal were identified only in ZH3 and ZK or had OAV values greater than 1, these compounds were considered to have caramel, fruity, and green aromas. In addition, although the OAVs of many volatile components in the three tea plant samples are all greater than 1, the OAVs of some compounds such as 1-octanol (with floral and fruity aroma), (E, Z)-2,6-nonadienal (with a fresh aroma), Octanal (with a citrus aroma), (E)-2-heptenal (with a fresh aroma), and hexanal (with a grassy aroma) vary significantly among different samples. This will lead to differences in aroma intensity and contribute to the shaping of the aroma characteristics of different tea samples (Yue et al., 2023).

**Table 1:** The OAV value of aroma-active volatiles, the OAV is greater than 1 in at least one sample.

ID	Name	CAS	Odor Description	OT(ug/L)	OAV		
					NB	ZH3	ZK
1	(Z)-3-Hexen-1-ol	544–12-7	fresh, green, grass, herbal	70	25.8	3.6	3.7
2	cis-Linalool oxide	5989-33-3	flowery sweet, woody	6	2.1	1.6	1.5
3	1-Octanol	111–87-5	rose-like or lemon-like	0.022	13,921.5	6139.4	6080.2
4	1-Nonanol	143–08-8	fresh, clean, fatty	0.0455	284.3	163.3	206.8
5	trans-nerolidol	40,716–66-3	floral, green, citrus	0.25	62.6	46.1	29.9
6	Linalool	78–70-6	lemon, citrus, orange, floral, sweet, woody	10	68.1	74.6	22.4
7	2,6-dimethyl-3,7-Octadiene-2,6-diol	51,276–34-7	flowery	1.38	3.8	1.3	NF
8	1-Octen-3-ol	3391-86-4	sweet	1	534.6	157.8	258.4
9	1-Pentanol	71–41-0	fusel-like, fruity	150.2	34.5	23.5	35.1
10	Phenylethyl Alcohol	60–12-8	floral, rose-like	390	1.1	0.5	0.6
11	2-Furanmethanol	98–00-0	burnt, sweet	4.5005	3.3	2.3	4.6
12	1-Hexanol	111–27-3	flower, alcoholic, sweet, oily, green, fruity	5.6	263.0	249.1	122.1
13	(E)-2-Hexenal	6728-26-3	almond, green, fruity,	110	0.8	2.1	1.3
14	(E,Z)-2,6-Nonadienal	557–48-2	fresh, cucumber-like	0.1	207.2	46.5	161.5
15	3-Methylbutanal	590–86-3	cocoa, almond	0.2	363.0	186.5	370.3
16	Methional	3268-49-3	cooked potato like	0.43	89.6	10.0	33.4
17	(E)-2-Nonenal	18,829–56-6	fatty, green	0.4	40.8	10.9	30.6
18	Octanal	124–13-0	citrus, green	0.52	118.0	33.2	94.3
19	(E)-2-Heptenal	18,829–55-5	soapy, pungent, fresh, almond, green, fatty	0.5	302.0	86.8	148.1
20	Nonanal	124–19-6	lemon, fishy, green, citrus	1.1	NF	33.4	77.8
21	(E,E)-2,4-Hexadienal	142–83-6	green, sweet, fruity, waxy, fatty	10	6.6	4.1	4.9
22	Butanal	123–72-8	musty, malt, bread, green, chocolate, cocoa	14	2.6	1.2	2.9
23	2-Methylbutanal	96–17-3	caramel musty, nutty	1	NF	206.6	335.6
24	Hexanal	66–25-1	grass, tallow, fat	2.4	274.9	22.1	86.0
25	Pentanal	110–62-3	grassy, bitter, pungent, metallic	8	473.6	180.2	487.4
26	2,6,6-Trimethyl-1,3-cyclohexadiene- 1-carboxaldehyde	116–26-7	woody, spicy	3	1.1	0.2	0.7
27	2,6,6-trimethyl-1-Cyclohexene-1-carboxaldehyde	432–25-7	fruity	3	4.4	0.6	0.8
28	Heptanal	111–71-7	green, oily, grassy	9.5	166.2	102.5	117.6
29	Hexanoic acid, ethyl ester	123–66-0	apple peel-like	1.16	30.0	3.9	6.6
30	Hexanoic acid, methyl ester	106–70-7	pine apple-like	10	3.3	1.3	1.4
31	Methyl salicylate	119–36-8	mint, wintergreen, peppermint	40	4.3	22.7	9.7
32	2-pentyl-Furan	3777–69-3	floral, fruit	6	28.0	10.1	18.8
33	trans-β-Ionone	79–77-6	floral	0.007	510.1	494.7	296.7
34	3-Methylene-1-oxa-spiro[4.5]decan-2-one	52,978–85-5	jasmine-like	0.007	303.2	NF	258.7
35	1-Hepten-3-one	2918-13-0	roasted, nutty	0.04	998.6	NF	NF
36	Jasmone	488–10-8	jasmine-like, herbal, floral, woody	1.9	9.0	4.6	2.7
37	4-Methyl-3-penten-2-one	141–79-7	honey-like, card board-like	200	3.4	3.8	3.2
38	6-Methyl-5-hepten-2-one	110–93-0	fruity, apple-like	50	3.0	2.1	2.3
39	trans-β-Ocimene	3779-61-1	warm, floral, herbal	200	3.0	0.1	0.3
40	β-Myrcene	123–35-3	balsam-like	15	2.1	0.9	2.2
41	Styrene	100–42-5	balsamic, gasoline	65	9.1	0.5	0.4
42	Indole	120–72-9	flower	11	6.8	3.2	3.1
43	Dimethyl sulfide	75–18-3	green	0.12	72,148.5	65,082.5	49,403.3
44	Naphthalene	91–20-3	strong mothball odor, dry, pungent, tarry	6	7.4	3.9	4.4

Note: NF stands for Undetected.

The odor characteristic and thresholds in water of relevant compounds have been described in Odor & Flavor Detection Thresholds in Water (μg/L) (http://www.leffingwell.com; http://www.chemicalbook.com; https://www.odor.org.uk); (Guo et al., 2021; H. Wang et al., 2022; Zhang et al., 2023).

## 4 Conclusions

In this study, we comprehensively revealed the varietal characteristics of three tea varieties, NB, ZH3, and ZK, by using multi-omics methods such as widely targeted metabolomics, HS-SPME-GC–MS, targeted metabolomics of amino acids, targeted metabolomics of anthocyanins, and HPLC quantitative analysis of catechins and caffeine. In the three tea plant samples, a total of 1770 non-volatile metabolites were identified, including a large number of substances such as flavonoids (265), polyphenols (131), terpenoids (227), and alkaloids (149). In the three groups of ZK vs NB, NB vs ZH3, and ZH3 vs ZK, 65, 55, and 90 differential metabolites (VIP > 1 and |log2(fold change)| > 1) were identified respectively. These differential metabolites were mainly components such as flavonoids and amino acids. The quantitative analysis of catechins, alkaloids and anthocyanins shows that the total contents of catechins and alkaloids in ZK tea are significantly higher than those in NB and ZH3. Although there is not much difference in the total content of anthocyanins among the three samples, the contents of most anthocyanins in ZK are also higher than those in NB and ZH3. However, the total amino acid content in ZK is much lower than that in ZH3 and NB. At the same time, 122 volatile compounds were identified, including 36 alcohols, 20 aldehydes, 16 esters, 16 ketones and other compounds. Overall, the content of volatile compounds in NB is significantly higher than that in ZH3 and ZK. In NB, ZH3 and ZK, 41, 36 and 37 key aroma-active volatiles (VIP > 1) were identified respectively. This study deeply reveals the unique quality foundation of albino tea, yellow-leaf tea, and purple-leaf tea, and provides a scientific basis for the further development of characteristic tea products and the improvement of tea processing techniques.

The following are the supplementary data related to this article.

## CRediT authorship contribution statement

**Yanlin An:** Writing – review & editing, Writing – original draft. **Lihong Zhang:** Software, Methodology. **Xueqi Li:** Supervision, Resources. **Xiaozeng Mi:** Validation. **Dahe Qiao:** Data curation. **Tingting Jing:** Project administration.

## Declaration of competing interest

The authors declare that they have no known competing financial interests or personal relationships that could have appeared to influence the work reported in this paper.

## Data Availability

Data will be made available on request.
